# Quantitative texture analysis comparison of three legumes

**DOI:** 10.3389/fpls.2023.1208295

**Published:** 2023-06-19

**Authors:** Rebekah Miller, Susan Duncan, Yun Yin, Bo Zhang, Jacob Lahne

**Affiliations:** ^1^ Department of Food Science and Technology, Virginia Tech, Blacksburg, VA, United States; ^2^ School of Plant and Environmental Sciences, Virginia Tech, Blacksburg, VA, United States

**Keywords:** texture, legume, vegetables, quality, compression, puncture

## Abstract

A validated texture-analysis method to evaluate product quality in frozen or cooked legumes is needed to support high-quality vegetable production but is not currently established in the literature. Peas, lima beans, and edamame were investigated in this study due to similar market use as well as growth in plant-based protein consumption in the United States. These three legumes were evaluated after three different processing treatments (blanch/freeze/thaw (BFT); BFT+microwave heat (BFT+M); BF+stove-top cooking (BF+C)), using both compression and puncture analysis following an American Society of Agricultural and Biological Engineers (ASABE) texture analysis method and moisture testing following an American Society for Testing and Materials (ASTM) standard method. Texture analysis results showed differences between legumes and processing methods. Compression analysis identified more differences between treatments within product type than puncture for both edamame and lima beans indicating compression might be more sensitive to texture changes in these products. Implementation of a standard texture method for legume vegetables for growers and producers would provide a consistent quality check to support efficient production of high-quality legumes. Due to the sensitivity obtained from the compression texture method in this work, compression should be considered for future research into a robust method to evaluate edamame and lima bean textures throughout the growing and production processes.

## 1 Introduction

Consumer acceptance, market value, and overall usefulness of food ingredients including vegetables and products are dependent on texture, which is highly influenced by growing and storage conditions as well as processing methods and techniques (Arntfield and Maskus, 2011). Properties of processed and raw fresh foods, such as legume vegetables, change over time due to microbial actions, oxidation, moisture migration and other factors, making accurate and complete understanding of the mechanical properties of foods, especially raw foods, difficult (Lu and Abbott, 2004). Quantification of quality attributes, such as texture, allows for a better understanding of the impact of changes occurring during production or storage of vegetable products prior to consumption. Additionally, plant foods, such as peas, lima beans, and vegetable soybean (*Glycine max* L. Merr.), commonly called edamame, have textural attributes based in their tissue structure, which could indicate textural differences between crops and other factors (Ilker and Szczesniak, 1990). Developing a method to quantify texture differences in vegetable legumes will result in a better understanding of the differences between varieties, growing conditions, postharvest factors and other variables experienced in agricultural production of plant foods.

Food texture measurements can be accomplished using destructive or non-destructive methods (Lu and Abbott, 2004). Destructive methods are more closely related to sensory evaluation methods, making destructive methods preferred over non-destructive methods, despite the limitation of collecting measurements from a sample instead of all products (Lu and Abbott, 2004). Of the many destructive methods, puncture and compression via texture analyzer can be used to understand the force required to penetrate and compress the food sample, respectively (Lu and Abbott, 2004). Texture research on convex, starchy vegetables, such as legumes, after processing is limited. Due to the varying consumption options of these products, puncture and compression methods are both relevant measurements to relate consumer consumption of peas, lima beans, and edamame to the analytical measurements.

Puncture analysis uses a probe smaller than the product being tested, resulting in the probe pushing through the surface and into the center of the product similar to a sensory experience of biting into the product using an incisor or canine tooth (Lu and Abbott, 2004). These results provide information on the force needed to break through the skin or outer surface of the product. Measurements can continue through the center and out the other side of the product resulting in a hole cored out of the sample. This provides measurements of force needed to penetrate both surfaces of the product.

Compression analysis uses a flat probe with enough surface area to fully cover the product being analyzed. The product is placed on a flat plate and as the probe comes into contact with the sample, the entire sample is flattened (Lu and Abbott, 2004; Wilhelm et al., 2004) similar to using molars to compress and consume a food. The force required to reach the point of rupture is measured. There is limited information on texture analysis through puncture or compression analysis of processed vegetable legumes such as peas, lima beans, and edamame or other vegetables of similar texture or use by manufacturers or consumers.

Demand for vegetable-sourced protein and protein products, including protein from legumes, has continued to diversify product options (Biddle, 2017). Frozen vegetables provide access to high-quality protein vegetable sources year-round. About 70% of the peas grown domestically are processed and frozen for sale (Biddle, 2017). Quality of vegetables and legume vegetables often refers to characteristics such as color, palatability, taste, size, and shape of the product (Biddle, 2017), all of which cause consumer inference on texture attributes resulting in overall appeal (Lawless and Heymann, 2010).

More than 70% of the edamame sold in the United States are imported from Asia (Yu et al., 2021) after being processed and frozen for distribution. Currently, edamame imported from Asia are of cultivars considered inferior in agronomic quality and consumer acceptability in the domestic market (Jiang et al., 2018). Domestic edamame crop production is a feasible addition for growers producing other bean crops and looking to diversify their field production to add economic value (Carson, 2010). With the growing consumer demand for edamame as a highly nutritional product, local farmers have an opportunity to grow and profit from edamame production (Garber and Neill, 2019). Carneiro et al. (2022) found consumers were willing to pay $0.77 more for dark green edamame beans compared to light green beans, indicating consumers are willing to pay more for what they perceive as higher quality edamame. Increasing the domestic production of edamame will result in fresh market potential for this vegetable and expand available product for incorporation of edamame into a variety of fresh and processed products. Recently released edamame varieties, such as VT Sweet (Zhang et al., 2021), are developed with consideration for quality and consumer acceptability, thus honed to the American palate. Edamame is harvested prior to full maturity, during the R6 development stage, when the pods are full of bright green beans (Fehr et al., 1971; Guo et al., 2020). However, harvest timing is challenging and can readily result in differences in edamame quality. Edamame product quality is measured on a combination of agronomic conditions and post-harvest characteristics such as sensory, and nutritional composition (Carneiro et al., 2020).

The popularity of edamame is increasing in the United States due to the nutritional value provided to consumers and economic value it can add to growers, producers, and processors of varying production capacity (Jiang et al., 2018; Garber and Neill, 2019). Edamame can be easily substituted for peas and lima beans (Klausner, 2004). All of these legumes can be added to salads, soups, stir-fries, or served as a side dish as an added component of flavor, texture, and nutrition. The versatility of these vegetables is a driving factor of their success in the market.

Peas and lima beans are already commonly produced in the United States. Peas are also grown and processed in Canada, France, China, and Russia and lima beans are also produced in Latin America and Canada (Arntfield and Maskus, 2011). As the edamame market and availability in the United States grow, quality specifications are needed for efficient production and higher consumer satisfaction. Many methods and approaches for texture analysis can be applied for specific purposes or products (Wilhelm et al., 2004). The American Society of Agricultural and Biological Engineers (ASABE S368.4) published a standard method for food materials with convex shape, such as edamame (ASAE, 2017a). Validating and standardizing a method specific to edamame texture will reduce variability in production and increase product quality in the market.

The goal of this study was to evaluate texture analysis methods for sensitivity to detect changes in texture of vegetable legumes. Texture analysis was completed in reference to three different preparation treatments (blanch/freeze/thaw (BFT) as a control; BFT+microwave heat (BFT+M); BF+stove-top cooking (BF+C)) and comparison of competitive protein-rich legume vegetables, peas, lima beans, and edamame, all of which have similar potential in salads, stir fries and other uses in the market (Klausner, 2004). Moisture was also evaluated as a driving force of texture making it appropriate to relate these attributes while understanding many additional factors could potentially impact texture (Ilker and Szczesniak, 1990). Application of ASABE S368.4 texture analysis method for vegetable legumes has previously been reported on lima beans (Aghkhani et al., 2012) but has not been used to evaluate peas or edamame. This applies the standard texture method to each product providing a previously unreported application and the ability to compare textures across product types.

## 2 Materials and methods

### 2.1 Product

Products of commercially processed (blanched and frozen) edamame, lima beans, and peas were purchased as shelled and frozen product from supermarkets (Krogers, Cincinnati, OH) in the local area (Blacksburg and Christiansburg, Virginia) (**Supplemental Materials Table 1**). Two lot numbers were obtained for each product brand. Two bags of each vegetable, brand, and lot number, except the Brand P lima beans, were purchased and transported in coolers with icepacks to the Virginia Tech Food Science and Technology building and stored in a freezer (True Manufacturing Company Inc., O’Fallon, Missouri) at -15°C prior to sample preparation and analysis.

### 2.2 Product preparation

Three separate treatments were tested: (1) blanch/freeze/thaw (BFT); (2) BFT+microwave (BFT+M) (3) BF+stove-top cooking (BF+C). BFT products were thawed at refrigeration temperature (2°C) in a refrigerator (True Manufacturing Company Inc., O’Fallon, Missouri) overnight prior to analysis. These blanched, frozen, and thawed (BFT) products did not undergo any additional heat treatment and served as the control treatment. Microwave heated (BFT+M) products were processed in a modified method seen in previous studies (Carneiro et al., 2021) to represent the typical cooking method. Products were allowed to thaw in refrigeration temperatures for eight to twelve hours, microwaved in 50-gram batches in a 1L Pyrex glass measuring container, covered with a paper towel, for forty seconds in a carousel microwave (model R-2W38, 120 VAC, 60Hz, 1200 watts, Sharp Corporation, Thailand), and refrigerated overnight. Carneiro et al. reported a similar process with microwaving occurring for 4min in polyethylene plastic bags (2021). This length of heating time was determined to be inappropriate in this study as only 50-gram batches of products were prepared at a time.

Cooked products (BF+C) were prepared following stovetop cooking instructions for the entire package contents (**Supplemental Materials Table 1**) on Brand B (pea) and Brand P packaging (edamame, lima bean). Pea products were put in a 2.7L pan (Tefal, Rumilly, Haute-Savoie, France) with 118.3 mL of water, covered with a lid and cooked over medium heat on a gas stove (Southbend, Fuquay Varina, NC) for 5 min., then removed from heat and allowed to stand, covered, for 2min. Peas were then drained and allowed to cool at room temperature. Lima beans were covered with water, brought to a boil for 3min, then covered and reduced to a simmer for 25min before draining and cooling. Edamame beans were covered with water and brought to a boil for 3min before draining and cooling. Listed instructions were applied by sample type to recreate the texture to be closest to appropriate and acceptable texture by the producers and consumers for each sample type. All product treatments were stored in the refrigerator for four to twenty-four hours prior to texture and moisture analyses to ensure consistent temperature across products and treatments. Though BF+C treatments varied by product, the preparation methods represented the intended product texture for each legume.

While peas, lima beans, and edamame can be consumed hot or cold, products were tested at refrigeration temperature to mimic the sensory attributes of items on a salad bar. The cool refrigeration temperature also allows greater control in product temperature during testing and relates to ‘salad bar conditions’ when comparing to sensory data.

### 2.3 Analysis

Two brands were used for each vegetable for replication with two lots from each brand (**Supplemental Materials Table 1**). Experiment was designed to compare 3 different vegetables (edamame, pea, lima bean) with 3 different preparation treatments (BFT; BFT+M; BF+C) from 2 different brands each with 2 lots within the brand and 20 beans, with minor exceptions (see **Supplemental Materials Table 2**) per treatment lot.

Puncture and compression testing were both completed following the ASABE S368.4, Compression Test of Food Materials of Convex Shape, guidelines using the TA XT Plus by Texture Technologies Corporation (Hamilton, MA) (**Supplemental Materials Figure 1**) (ASAE, 2017a). Lima beans and edamame were oriented horizontally on the texture analyzer surface in reference to the hilum for consistency; though results will vary depending on vertical or horizontal orientation, one is not superior to the other (Paulsen, 1978). Products were positioned under the probe to ensure the tallest part of the vegetable made contact with the center of the probe. This alignment reduced sample movement and breakage during testing before the point of rupture was reached. Products with broken skin or damaged exteriors were excluded from the study. For puncture testing, beans were tested individually, arranged on a plate with hole, allowing the probe to completely penetrate the vegetable calculating surface strength as the probe entered (force 1) and exited (force 2) the sample. For compression testing, each individual bean was tested with the force-deformation curve recorded through the point of rupture. Puncture and compression analysis aimed to analyze twenty individual beans from each lot and treatment. Puncture testing used a 2mm puncture probe moving at 2.00mm/min. Compression used a flat plat probe moving at 2.00mm/min with 70% strain.

Moisture content of each sample and treatment was determined based on the American Society for Testing and Materials (ASTM) standard method S352.2 designed to measure moisture of unground grains and seeds (ASAE, 2017b). Legumes were prepared following the processing described and weighed (15 g). Testing was completed in triplicate. Products were placed in a hot air-drying oven (model OV702F, Thermo Scientific, Waltham, MA) at 105°C until a consistent weight was reached at approximately 72 hrs. Products were then brought to room temperature in a desiccator before being weighed. Products were weighed again, and moisture was calculated by dividing the sample weight loss (g) by the original sample weight (g) and multiplying by 100 ( $\frac{loss\;in\;weight\;\left(g\right)}{inital\;weight\;\left(g\right)}\times 100$ ) (ASAE, 2017b).

Statistical analysis of puncture, compression, and moisture data was completed using JMP Pro 15 (SAS, Cary, NC). Mixed model ANOVA was conducted followed by Tukey’s HSD.

## 3 Results

### 3.1 Puncture

**Figure 1:**
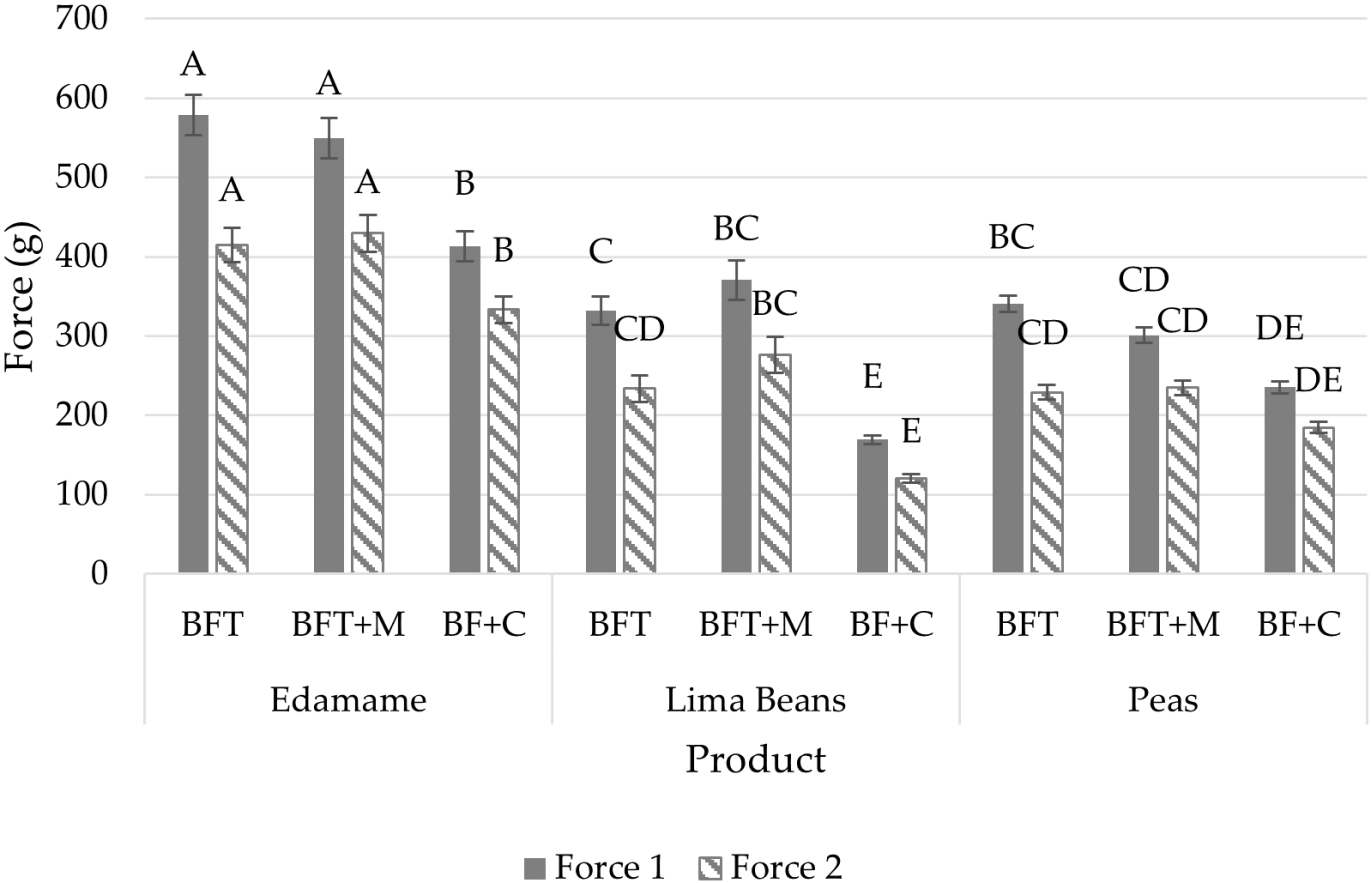
Puncture results (mean) of force 1 (g) and force 2 (g) by product (edamame; lima beans; peas) and treatment (blanch/freeze/thaw (BFT); BFT+microwave (BFT+M); BF+stove-top cooking (BF+C)). Error bars were constructed using 1 standard error from the mean. Tukey's HSD connecting letters indicate similarities within force 1 and force 2 respectively and were calculated with a fixed effects model.

Mixed model ANOVA of these results showed significant interaction between product type and treatments in both force 1 (p<0.05) and force 2 (p<0.05). Tukey’s HSD results (**Figure 1**) of a fixed model ANOVA show the BFT and BFT+M treatments to have similar impacts on each product type while the BF+C treatment reduced the force required for puncture which is likely due to structural changes which occur during cooking such as cell breakdown and start gelatinization.

Edamame required more force to puncture the product across treatments with overall results of force 1 at 513.45 ± 14.30 (mean ± SE (g)) and force 2 at 392.62 ± 12.26 (mean ± SE (g)) as compared to the lima beans with force 1 at 290.68 ± 11.79 (mean ± SE (g)) and force 2 at 210.10 ± 10.40 (mean ± SE (g)) and peas with force 1 at 292.12 ± 6.00 (mean ± SE (g)) and force 2 at 216.07 ± 5.12 (mean ± SE (g)) indicating edamame is a firmer legume compared to peas and lima beans (14) (**Figure 1**).

Puncture analysis was unable to differentiate between BFT and BFT+M treatments of the three products from force 1 or force 2. However, puncture results indicated higher forces required by edamame than lima beans and peas in all cooking treatments (BFT, BFT+M, BF+C).

### 3.2 Compression

**Figure 2:**
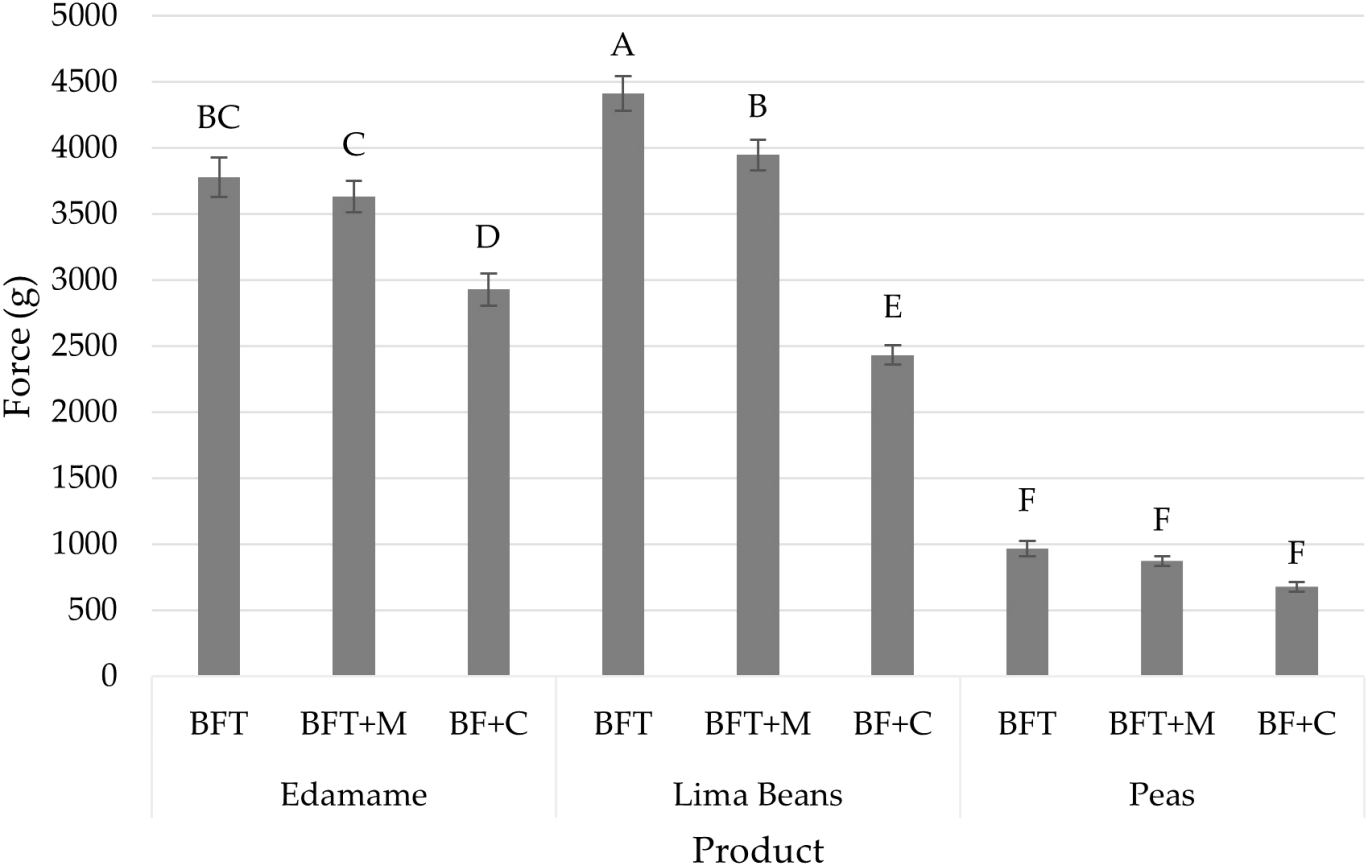
Compression results (mean) of force (g) by product (edamame; lima beans; peas) and treatment (blanch/freeze/thaw (BFT); BFT+microwave (BFT+M); BF+stove-top cooking (BF+C)). Error bars were constructed using 1 standard error from the mean. Tukey's HSD connecting letters indicate similarities and were calculated with a fixed effects model.

Mixed model ANOVA showed significant interaction between product type and treatments (p<0.05). Tukey’s HSD results (**Figure 2**) of a fixed model ANOVA showed each treatment of peas to be similar texture among the product type while the treatments of edamame and lima beans showed similarities based on treatment type within the two products. Across treatments, peas required less force to cause sample rupture, with an overall mean force of 837.25 ± 68.59 (mean ± SE (g)), than both edamame and lima beans which required 3402.08 ± 68.02 (mean ± SE (g)) and 3598.94 ± 68.02 (mean ± SE (g)) respectively. These force values indicated that processed peas persisted less hardness than both processed edamame and lima beans (Lu and Abbott, 2004).

Compression analysis differentiated between treatments implemented for both edamame and lima beans but was unable to differentiate any of the treatments of peas. All treatments across peas (BFT, BFT+M, BF+C) were found to be similar in Tukey’s HSD connecting letters.

### 3.3 Moisture

**Figure 3:**
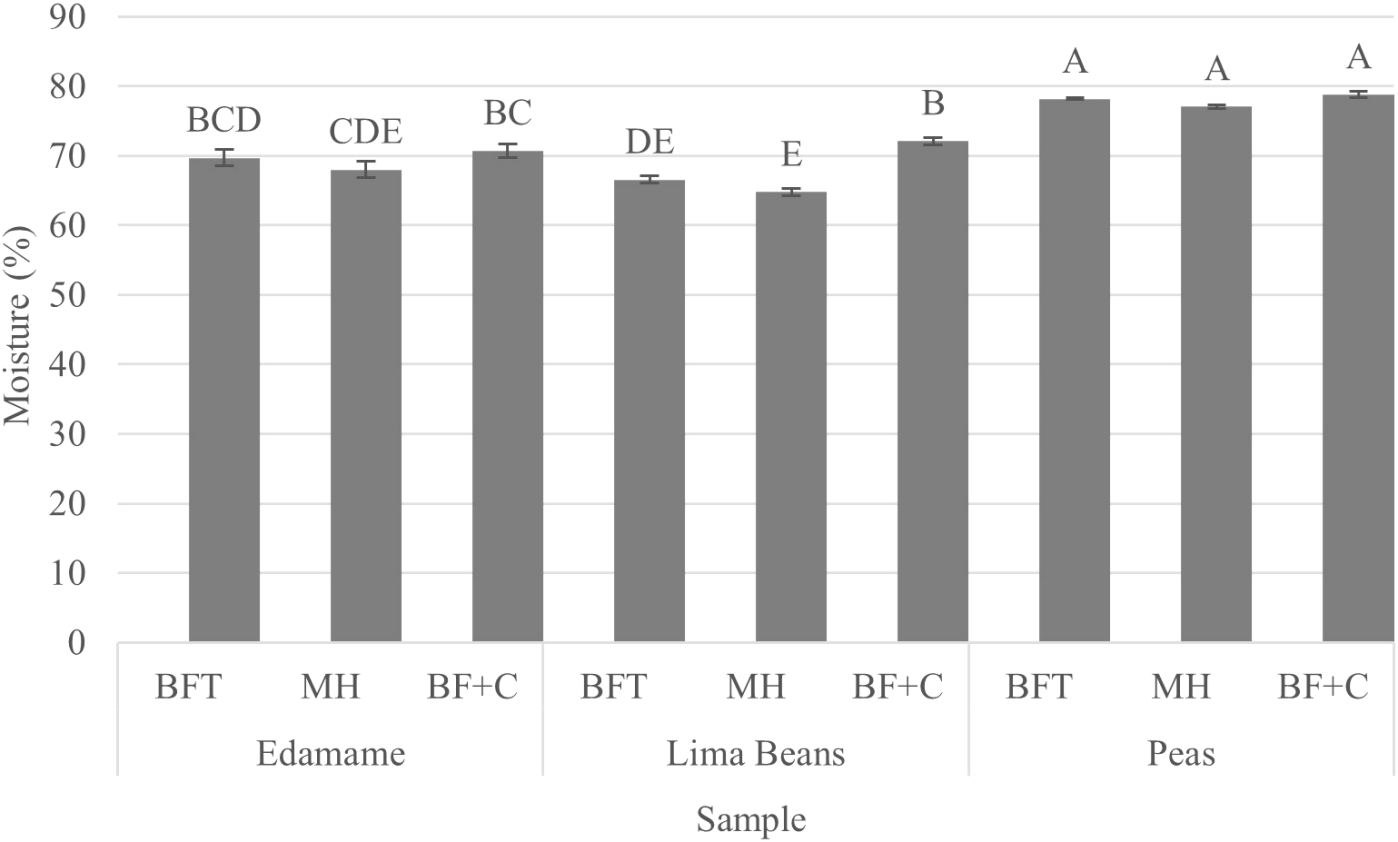
Results (mean) of moisture (%) by product (edamame; lima beans; peas) and treatment (blanch/freeze/thaw (BFT); BFT+microwave (BFT+M); BF+stove-top cooking (BF+C)). Error bars were constructed using 1 standard error from the mean. Tukey's HSD connecting letters indicate similarities and were calculated with a fixed effects model.

Mixed model ANOVA of the moisture results showed significant interaction between sample type and treatments (p<0.05). Results showed both sample type (p<0.05) and treatments (p<0.05) to have at least one difference. Tukey’s HSD results showed vegetables were similar within sample type except lima beans which showed higher moisture content in the BF+C treatment group (**Figure 3**). Across treatments, peas had higher moisture content at 78.00 ± 0.53 (mean ± SE (%)) than both edamame and lima beans at 69.47 ± 0.53 (mean ± SE (%)) and 67.82 ± 0.53 (mean ± SE (%)) respectively. This difference may explain the lower compression force required to rupture the products when compared to edamame and lima beans products.

## 4 Discussion

Though edamame, peas, and lima beans all have similarities in use and interest in the domestic market, texture of these vegetable legumes vary based on product type and preparation. Mean force required to puncture through edamame products were consistently higher than both lima beans and peas. Using compression, peas required less force to rupture compared to edamame and lima beans. Based on our results, generally, lima beans and peas are more similar to each other than edamame based on puncture while edamame and lima beans are more similar to each other than peas through compression.

Compression analysis more successfully differentiated between treatments implemented for both edamame and lima beans. However, compression was unable to differentiate between any of the implemented treatments of peas. Puncture analysis was more successful differentiating the processing treatments of peas but was not precise enough to fully differentiate each treatment entirely. This was also true for the BFT and BFT+M treatments of edamame and lima beans. Puncture analysis was able to differentiate the BF+C treatments of edamame and lima beans, however.

BF+C treatment methods varied by product to better evaluate the intended state of each individual product. This variation limits the ability to compare this treatment method across product types while also providing a baseline specific to each product. Differences across products within the BF+C treatments show the presumably intended texture for these products as established by the brands. The BF+C treatment also consistently required less force for each legume over BFT and BF+M with edamame and lima beans showing this with statistical significance. This variation from edamame and lima beans to peas is likely due to not only the intrinsic structure differences between legumes, but also the longer cooking time required to prepare these products, which caused structural changes such as further protein denaturation or breakdown of starch granules caused by heat.

Total starch content and fibre in legumes varies by legume type and variety. Edamame nutritional content also varies by maturity at harvest (Yu et al., 2021). Nikolopoulou et al. found variety to impact nutrition of peas as well as environmental factors and growing year (2007). It is likely that variety, environment, year, and location can also impact nutritional content and other attributes of crops including legume vegetables.

When harvested at optimal maturity, Yu et al. found edamame to be around 12% starch and 6% fibre while noting higher starch content in edamame is often preferred to achieve softer edamame after cooking caused by starch gelatinization as well as pectin solubilization (2021). Lima beans tend to be higher in starch with 35-40% starch and 6-7% fibre on a wet basis reported while peas have the highest starch of these three legumes with 55-68% starch and 3-7% fibre reported also on a wet basis (Arntfield and Maskus, 2011). These numbers help explain the low forces required for both puncture and compression of peas. As they have the highest starch content, they would also likely soften due to starch gelatinization during any heat process. As the compression data of peas shows the force required for each processing method is the lowest of all vegetables, peas also have the highest moisture contents. This moisture content likely contributes to the low forces required to reach the rupture point as the water present in the sample could provide less resistance to the probe than the starch, protein and fibre that are more prominent in the edamame and lima beans. These patterns may change when analysing samples which were not previously frozen and is a path for additional research in this area. Additional research into a standardized texture analysis for legume vegetables, fresh or frozen, will be needed to conclude either of these methods to be appropriate for implementation into quality control programs.

While lima beans and peas both have a thicker skin around the starchy centre holding the vegetable together and creating contrast in texture, edamame was observed to have less texture variation through the structure of the bean. Though this observed lack of texture contrast was not measured in this work, the observation helps explain additional differences among the products researched. The methods employed in this researched does not specifically measure this characteristic however, these similarities and differences may be considered when selecting ingredients for a new or reformulated food or food product.

Our knowledge around the vegetable growing and processing conditions including varieties produced, processing by brand, and vegetable type is a significant limitation of this research. Variations in data by lot within brand for lima beans and peas are not easily explained as physical appearance of products and moisture results do not support any obvious conclusions. These variations between lots within sample types and brands maybe due to differences in growing and/or processing conditions that are not known by the researchers in this study. Edamame products had statistical differences in results of brand 1 (Brand P) edamame lots which were likely due to poor quality of edamame in the lot (L680). Half of the edamame in the packages were yellow, indicating late harvest of the product which also implies lower moisture content compared to edamame harvested on time. The higher force required, and lower moisture content of these products confirm their maturity and inferior quality initially inferred based on the vegetable colour. Continuing this research with additional brands or products grown and processed in controlled environments may result in a better understanding of method outcomes as related to the growing and processing conditions of which the products were subjected.

The results of this study do not relate to any product evaluation of warm product. Storage and analysis at refrigeration temperature was chosen to reduce variability in analysis and relates directly to cold consumption uses of these products such as how they are on a salad bar or other cold foods. Consumer perception of products will vary based on temperature due to influence on flavour perception and needs to be considered for research relating to consumers.

Development and implementations of an instrumental-based texture method and quality standards for legumes quality control readily adopted by growers and producers are of vital importance for plant-based market. This study showed compression texture analysis could be useful when determining maturity of edamame as well as processing changes in edamame and lima beans. Setting standards could more easily guide and determine appropriate quality specifications for products through the processing plans.

For texture testing by growers and producers, the compression method may be more sensible for implementation due to the ease of data analysis and relation to chewing with molars as these products would often be crushed in the mouth more than punctured. Additionally, puncture analysis was not able to distinguish processing differences in edamame, lima beans, or peas while compression analysis was sensitive enough to detect these distinctions in edamame and lima beans.

Continuing research focused on the compression method researched here would be advised over the puncture method due to the ease of analysis and sensitivity of analysis for both edamame and lima beans. While the puncture method results in two forces, the compression method gives a single force output. Puncture analysis was able to distinguish treatments of peas better than compression analysis, but this was not true of lima beans and edamame. However, this work has shown utilizing the ASABE S368.4, Compression Test of Food Materials of Convex Shape, is an effective method which can be applied further to determine specific standard methods for legumes (ASAE, 2017a).

Additional research should be completed to better relate this resulting force to texture attributes. Specific quality parameters would need to be established based on the individual product, desired quality and sensory attributes, moisture content, and other variations among vegetables, locations, and facilities. Cooking methods impact product characteristics differently due to unique product attributes. Texture changes can also be seen due to storage conditions and time, moisture migration, and general degradation of the food structure throughout the processing and shelf life.

## Data Availability

The original contributions presented in the study are included in the article/**Supplementary Material**. Further inquiries can be directed to the corresponding author.
