# Corrigendum: Quantitative texture analysis comparison of three legumes

**DOI:** 10.3389/fpls.2024.1490251

**Published:** 2024-11-13

**Authors:** Rebekah Miller, Susan Duncan, Yun Yin, Bo Zhang, Jacob Lahne

**Affiliations:** ^1^ Department of Food Science and Technology, Virginia Tech, Blacksburg, VA, United States; ^2^ School of Plant and Environmental Sciences, Virginia Tech, Blacksburg, VA, United States

**Keywords:** texture, legume, vegetables, quality, compression, puncture

In the published article, there was an error in **Figure 1**, **Figure 2**, and **Figure 3** as published. The error bars included in the figures were incorrect. The corrected Figures and captions appear below.

**Figure 1:**
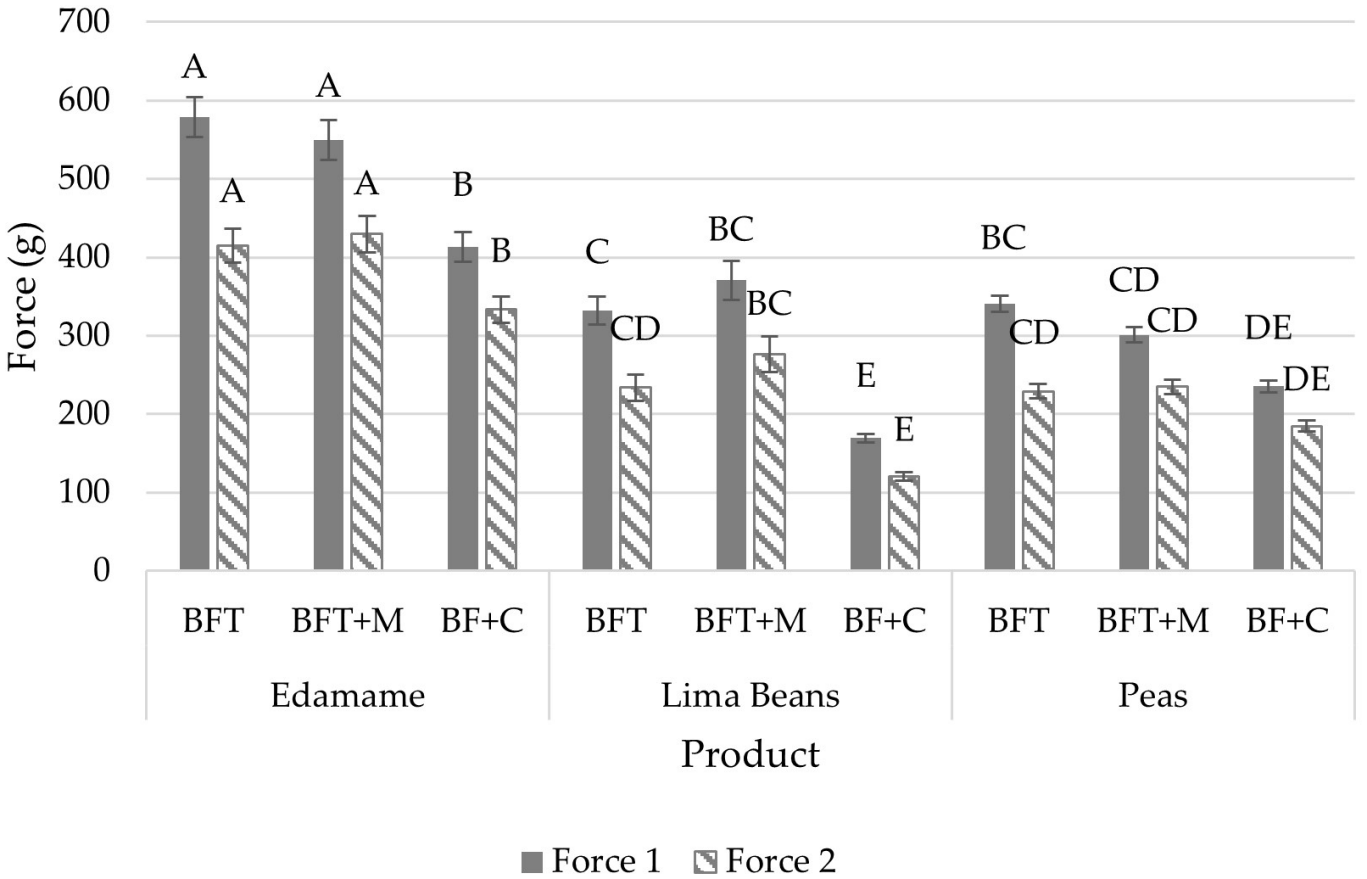
Puncture results (mean) of force 1 (g) and force 2 (g) by product (edamame; lima beans; peas) and treatment (blanch/freeze/thaw (BFT); BFT+microwave (BFT+M); BF+stove-top cooking (BF+C)). Error bars were constructed using 1 standard error from the mean. Tukey’s HSD connecting letters indicate similarities within force 1 and force 2 respectively and were calculated with a fixed effects model.

**Figure 2:**
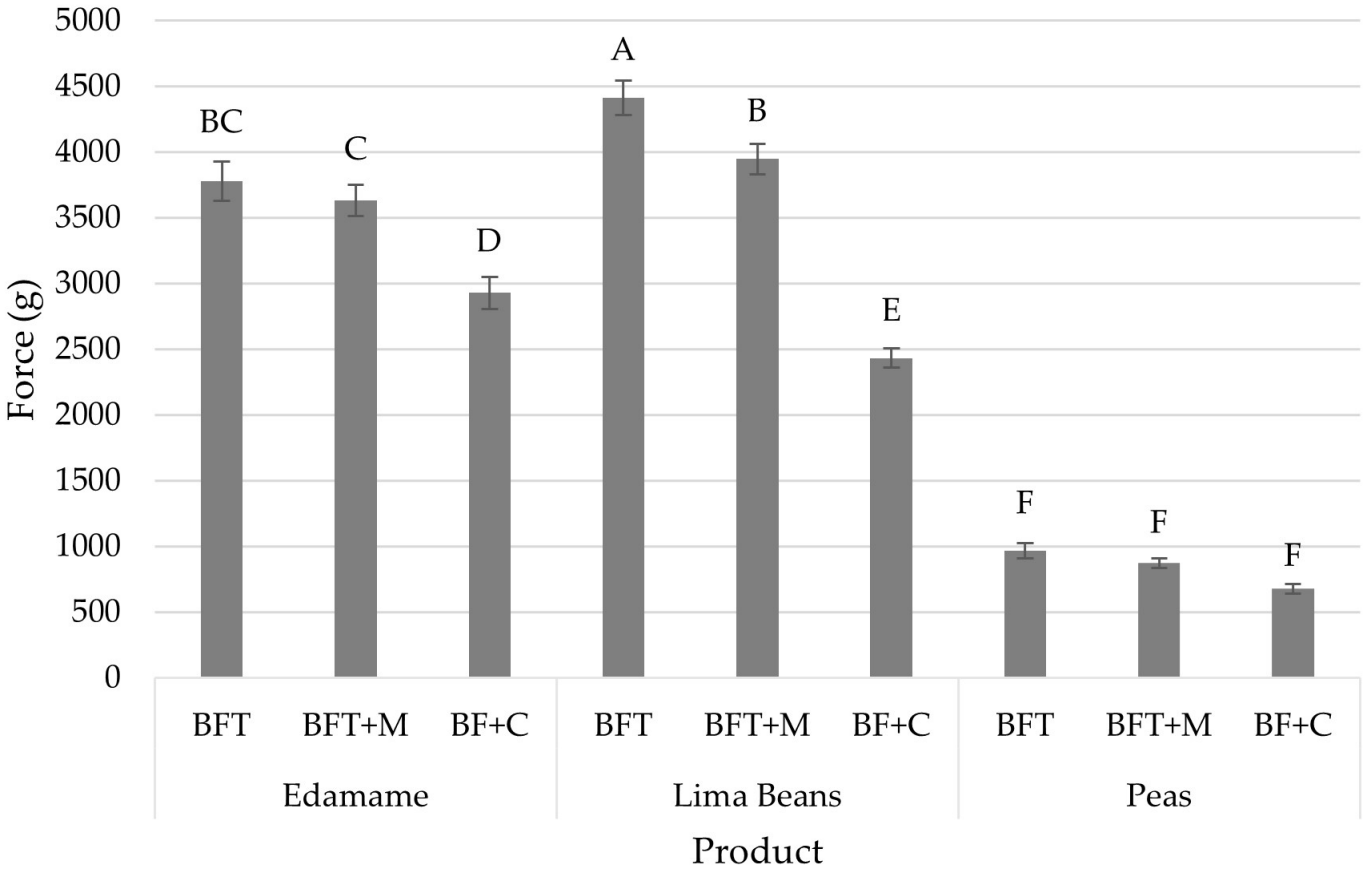
Compression results (mean) of force (g) by product (edamame; lima beans; peas) and treatment (blanch/freeze/thaw (BFT); BFT+microwave (BFT+M); BF+stove-top cooking (BF+C)). Error bars were constructed using 1 standard error from the mean. Tukey’s HSD connecting letters indicate similarities and were calculated with a fixed effects model.

**Figure 3:**
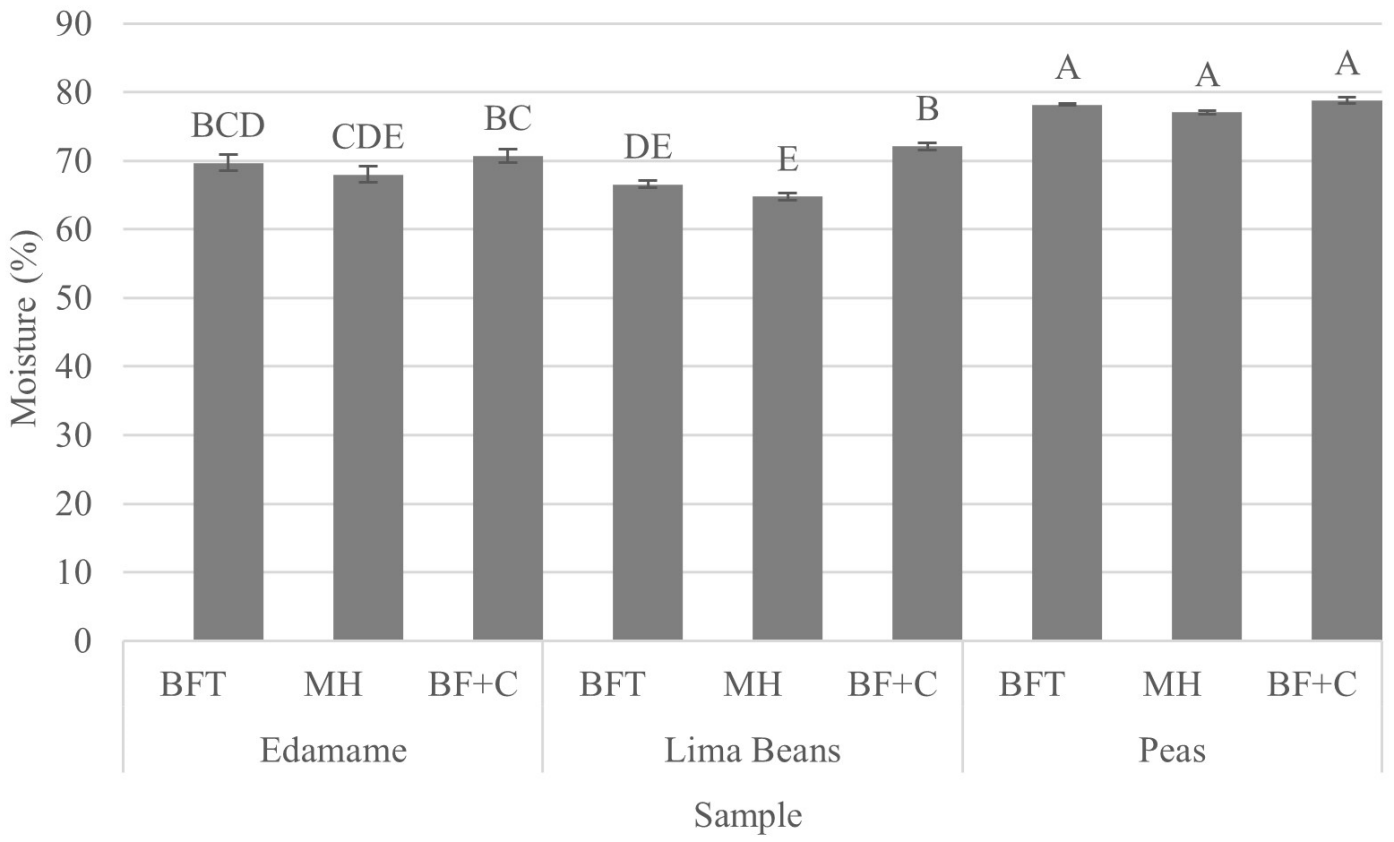
Results (mean) of moisture (%) by product (edamame; lima beans; peas) and treatment [blanch/freeze/thaw (BFT); BFT+microwave (BFT+M); BF+stove-top cooking (BF+C)]. Error bars were constructed using 1 standard error from the mean. Tukey’s HSD connecting letters indicate similarities and were calculated with a fixed effects model.

The authors apologize for this error and state that this does not change the scientific conclusions of the article in any way. The original article has been updated.

