# Supplementary material for: Quantitative texture analysis comparison of three legumes
Source: Front Plant Sci. 2023 Jun 19;14:1208295. doi: 10.3389/fpls.2023.1208295 (PMC10316706; doi:10.3389/fpls.2023.1208295)
Supplement: Supplementary file 1 [file DataSheet1.docx]

Supplementary Material

Quantitative Texture Analysis Comparison of Three Legumes

**Rebekah Miller^*^, Susan Duncan, Yin Yun, Bo Zhang, Jacob Lahne**

*** Correspondence:** Rebekah Miller: rebekahm20@vt.edu

#
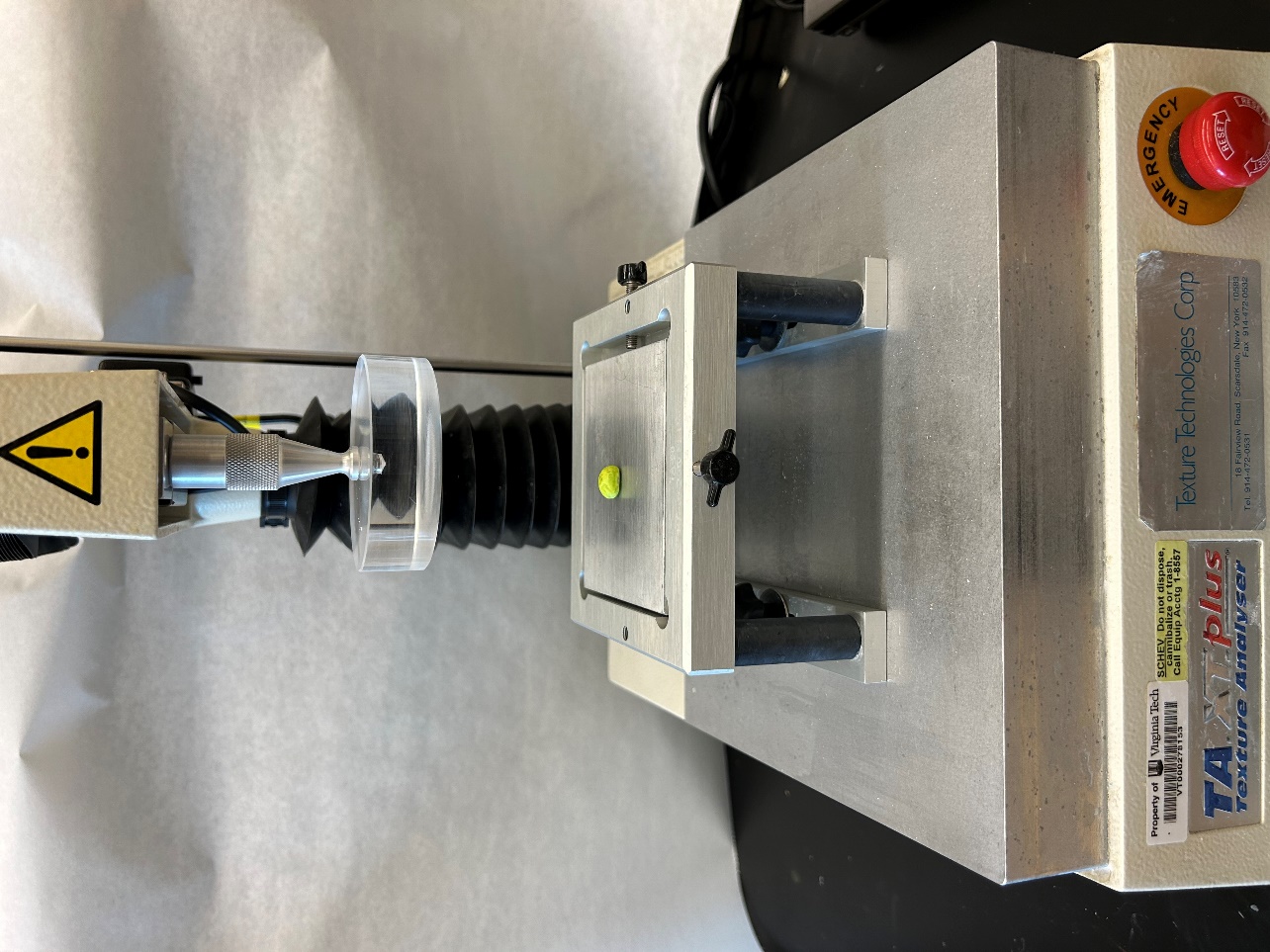
Supplementary Figure

**Supplementary Figure 1.** TA XT Plus texture analyzer by Texture Technologies Corporation (Hamilton, MA) used in this study fitted with the compression probe and edamame bean in place for analysis.
