# Supplementary material for: Quantitative texture analysis comparison of three legumes
Source: Front Plant Sci. 2023 Jun 19;14:1208295. doi: 10.3389/fpls.2023.1208295 (PMC10316706; doi:10.3389/fpls.2023.1208295)
Supplement: Supplementary file 2 [file Table1.docx]

Supplementary Material

Quantitative Texture Analysis Comparison of Three Legumes

**Rebekah Miller^*^, Susan Duncan, Yin Yun, Bo Zhang, Jacob Lahne**

*** Correspondence:** Rebekah Miller: rebekahm20@vt.edu

# Supplementary Data

| **Sample Type** | **Brand 1** | | | | **Brand 2** | | | |
| --- | --- | --- | --- | --- | --- | --- | --- | --- |
|  | **Brand Name*** | **Lot Number*** | **DNW** | **Best By Date** | **Brand Name*** | **Lot Number*** | **DNW** | **Best By Date** |
| Peas | Brand S^1^ | L737^1^ | 340g | Apr 2023 | Brand B^2^ | L520^1^ | 283.5g | Sept 1, 2022 |
|  |  | L454^2^ | 340g | Apr 2023 |  | L420^2^ | 283.5g | Sept 30, 2022 |
| Lima Beans | Brand K^1^ | L161^1^ | 340g | Oct 26, 2022 | Brand P^2^ | L031^1^ | 567g | Apr 13, 2023 |
|  |  | L991^2^ | 340g | Oct 9, 2022 |  | L661^2^ | 567g | Jun 15, 2023 |
| Edamame | Brand P^1^ | L680^1^ | 227g | Jun 16, 2022 | Brand S^2^ | L452^1^ | 340g | Apr 2023 |
|  |  | L871^2^ | 227g | Jul 6, 2023 |  | L890^2^ | 340g | Apr 2023 |

Table 1. Product information of samples used in the study. ^*^Brand identity and lot number has been coded for this study. ^1^Notates brand or lot 1 as appropriate. ^2^Notates brand or lot 2 as appropriate.
