# Supplementary material for: Quantitative texture analysis comparison of three legumes
Source: Front Plant Sci. 2023 Jun 19;14:1208295. doi: 10.3389/fpls.2023.1208295 (PMC10316706; doi:10.3389/fpls.2023.1208295)
Supplement: Supplementary file 3 [file Table2.docx]

Supplementary Material

Quantitative Texture Analysis Comparison of Three Legumes

**Rebekah Miller^*^, Susan Duncan, Yin Yun, Bo Zhang, Jacob Lahne**

*** Correspondence:** Rebekah Miller: rebekahm20@vt.edu

| Cooking Method | Brand | Edamame | | | | | | Lima Beans | | | | | | Peas | | | | | |
| --- | --- | --- | --- | --- | --- | --- | --- | --- | --- | --- | --- | --- | --- | --- | --- | --- | --- | --- | --- |
|  | | **Compression (Force(g))** | | | | | | | | | | | | | | | | | |
|  |  | Lot 1 | | | Lot 2 | | | Lot 1 | | | Lot 2 | | | Lot 1 | | | Lot 2 | | |
|  |  | mean | ± | SE | mean | ± | SE | mean | ± | SE | mean | ± | SE | mean | ± | SE | mean | ± | SE |
| BFT | 1 | 4158.96 | ± | 330.14 | 2789.59 | ± | 156.39 | 4913.60 | ± | 259.39 | 5096.65 | ± | 235.67 | 1184.51 | ± | 94.36 | 903.06 | ± | 149.73 |
|  | 2 | 4041.92 | ± | 236.13 | 4134.36 | ± | 328.01 | 3641.53 | ± | 192.75 | 4019.05 | ± | 228.57 | 917.99 | ± | 105.03 | 851.94 | ± | 77.33 |
| BFT+M | 1 | 4057.37 | ± | 240.20 | 2663.99 | ± | 176.54 | 4574.49 | ± | 192.64 | 3969.20 | ± | 265.73 | 1015.37 | ± | 60.05 | 699.74 | ± | 76.34 |
|  | 2 | 3606.32 | ± | 205.97 | 3643.73 | ± | 223.76 | 3721.71 | ± | 147.65 | 3530.43 | ± | 249.66 | 911.61 | ± | 67.47 | 870.82 | ± | 67.98 |
| BF+C | 1 | 3282.18 | ± | 246.18 | 1994.95 | ± | 108.21 | 2427.86 | ± | 95.49 | 2847.56 | ± | 138.23 | 908.34 | ± | 63.17 | 519.30 | ± | 31.85 |
|  | 2 | 3039.67 | ± | 188.09 | 3411.94 | ± | 273.74 | 2322.14 | ± | 131.88 | 2127.88 | ± | 170.33 | 777.19 | ± | 92.89 | 509.94 | ± | 56.59 |
|  | | **Puncture (Force 1 (g))** | | | | | | | | | | | | | | | | | |
| BFT | 1 | 695.94 | ± | 60.16 | 345.59 | ± | 21.69 | 319.61 | ± | 38.72 | 411.17 | ± | 52.77 | 403.46 | ± | 20.25 | 283.90 | ± | 19.59 |
|  | 2 | 630.25 | ± | 40.36 | 641.50 | ± | 34.92 | 319.87 | ± | 22.28 | 277.18 | ± | 13.58 | 344.38 | ± | 17.55 | 328.77 | ± | 16.20 |
| BFT+M | 1 | 645.36 | ± | 62.98 | 345.68 | ± | 20.16 | 353.40 | ± | 43.17 | 448.17 | ± | 56.94 | 341.61 | ± | 21.26 | 259.03 | ± | 17.38 |
|  | 2 | 576.67 | ± | 34.11 | 625.34 | ± | 45.94 | 327.43 | ± | 51.85 | 353.41 | ± | 44.54 | 311.17 | ± | 17.84 | 292.02 | ± | 15.25 |
| BF+C | 1 | 420.83 | ± | 44.21 | 277.20 | ± | 61.97 | 164.36 | ± | 13.41 | 192.14 | ± | 13.10 | 257.81 | ± | 12.54 | 247.33 | ± | 16.61 |
|  | 2 | 496.85 | ± | 39.22 | 456.41 | ± | 30.96 | 154.96 | ± | 4.91 | 166.09 | ± | 7.76 | 236.27 | ± | 17.68 | 199.61 | ± | 12.93 |
|  |  |  |  |  |  |  |  |  |  | **Puncture (Force 2 (g))** | | | |  |  |  |  |  |  |
| BFT | 1 | 556.54 | ± | 52.72 | 278.40 | ± | 26.01 | 234.29 | ± | 28.50 | 299.26 | ± | 54.49 | 263.85 | ± | 23.94 | 197.32 | ± | 12.61 |
|  | 2 | 459.30 | ± | 35.87 | 641.50 | ± | 26.70 | 230.81 | ± | 19.53 | 171.64 | ± | 9.91 | 211.17 | ± | 19.39 | 244.23 | ± | 16.13 |
| BFT+M | 1 | 573.74 | ± | 56.35 | 268.70 | ± | 29.08 | 272.82 | ± | 33.17 | 369.92 | ± | 68.62 | 263.45 | ± | 23.53 | 229.47 | ± | 10.06 |
|  | 2 | 464.37 | ± | 43.28 | 412.38 | ± | 28.65 | 220.63 | ± | 33.94 | 240.76 | ± | 28.88 | 210.80 | ± | 17.66 | 234.66 | ± | 14.63 |
| BF+C | 1 | 409.38 | ± | 38.99 | 196.97 | ± | 20.28 | 112.96 | ± | 10.40 | 142.45 | ± | 13.17 | 204.27 | ± | 14.67 | 201.39 | ± | 12.54 |
|  | 2 | 381.35 | ± | 30.63 | 346.23 | ± | 20.48 | 116.35 | ± | 8.11 | 109.33 | ± | 9.22 | 155.16 | ± | 13.42 | 177.07 | ± | 15.15 |
|  |  |  |  |  |  |  |  |  |  |  |  |  |  |  |  |  |  |  |  |
|  | | **Moisture (%)** | | | | | | | | | | | | | | | | | |
| BFT | 1 | 63.20 | ± | 0.31 | 73.25 | ± | 0.48 | 65.770 | ± | 0.31 | 64.40 | ± | 0.25 | 78.40 | ± | 0.22 | 78.37 | ± | 0.28 |
|  | 2 | 71.19 | ± | 0.45 | 71.16 | ± | 0.59 | 68.498 | ± | 0.63 | 67.62 | ± | 0.86 | 77.97 | ± | 0.35 | 77.93 | ± | 0.17 |
| BFT+M | 1 | 61.77 | ± | 0.38 | 71.09 | ± | 0.29 | 62.811 | ± | 0.22 | 63.98 | ± | 0.37 | 77.21 | ± | 0.25 | 77.60 | ± | 0.24 |
|  | 2 | 70.24 | ± | 0.12 | 68.95 | ± | 0.39 | 65.965 | ± | 0.82 | 66.22 | ± | 0.72 | 75.94 | ± | 0.15 | 77.50 | ± | 0.42 |
| BF+C | 1 | 65.09 | ± | 0.25 | 74.07 | ± | 0.29 | 71.62 | ± | 0.33 | 69.85 | ± | 0.12 | 78.27 | ± | 0.13 | 78.60 | ± | 0.32 |
|  | 2 | 71.85 | ± | 0.47 | 71.80 | ± | 0.51 | 72.63 | ± | 0.41 | 74.33 | ± | 0.44 | 77.09 | ± | 0.31 | 81.15 | ± | 0.20 |

Table 2. Means (+/- SE) for compression (force (g)), puncture (force 1 (g); force 2 (g)), and moisture (%) evaluation of three vegetables (edamame, lima beans, peas) as affected by preparation method (blanched, frozen, thawed (BFT); BFT+microwave (BFT+M); BF+cooking (BF+C)) by lot and replication.

^1^Edamame Brand 1: Brand P (lot 1:L680; lot 2:L871), Brand 2: Brand S (lot 1: L452; lot 2: L890); Lima Beans Brand 1: Brand K (lot 1: L161; lot 2: L991), Brand 2: Brand P (lot :L031; lot 2:L661); Peas Brand 1: Brand S (lot 1: L737; lot2: L454), Brand 2: Brand B (lot 1: L520; lot 2: L420). ^2^ The BFT peas during compression analysis only collected 19 data points from lots L737 and L520 and only 18 from L420. Similarly, the MH lima beans compression only collected 19 data points from lot L161. The puncture testing of edamame collected an extra data point resulting in a total of 21 from the BFT+M treatment of L890. These variations were due to data transfer issues. ^3^blanch/freeze/thaw (BFT); BFT+microwave heat (BFT+M); BF+stove-top cooking (BF+C).
